# Spatial distribution of diphtheria cases during the 2022/2023 outbreak in Kano State, Northern Nigeria

**DOI:** 10.1371/journal.pone.0351150

**Published:** 2026-06-25

**Authors:** Rayyan M. Garba, Zainab A. Umar, Zainab Abdulkadir, Usman Bashir, Rabiu I. Jalo, Yasir N. Jibril, Aminu A. Yusuf, Kabiru I. Gurama, Abdullahi K. Suleiman, Zainab U. Ibrahim, Sherifah Sheriff, Ibrahim D. Muhammad, Chizoba Okolo, Adetayo Aborisade, Abdulgafar L. Olawumi, Kabiru Abdulsalam, Nafisatu Bello-Muhammad, Umma A. Ibrahim, Oiza O. Aliu-Isah, Mustapha A. Yusuf, Aisha A. Galadanci, Jibrin Gambo, Yusuf A. Yusuf, Aishatu L. Adamu, Baba M. Musa, Muktar H. Aliyu

**Affiliations:** 1 Department of Community Medicine, Aminu Kano Teaching Hospital and Bayero University, Kano, Nigeria; 2 Department of Family Medicine, Aminu Kano Teaching Hospital, Kano, Nigeria; 3 Department of Otorhinolaryngology, Aminu Kano Teaching Hospital and Bayero University, Kano, Nigeria; 4 Department of Haematology and Blood Transfusion, Aminu Kano Teaching Hospital, Kano, Nigeria; 5 Center for Infectious Diseases Research, Bayero University, Kano, Nigeria; 6 Department of Paediatrics, Aminu Kano Teaching Hospital and Bayero University, Kano, Nigeria; 7 Department of Chemical Pathology, Aminu Kano Teaching Hospital & Bayero University, Kano, Nigeria; 8 Department of Obstetrics and Gynaecology, Aminu Kano Teaching Hospital and Bayero University, Kano, Nigeria; 9 Faculty of Dentistry, Aminu Kano Teaching Hospital, Kano and Bayero University, Kano, Nigeria; 10 Department of Medical Microbiology and Parasitology, Aminu Kano Teaching Hospital and Bayero University, Kano, Nigeria; 11 Department of General Studies, Bilyamin Usman Polytechnic, Hadejia, Nigeria; 12 Department of Environmental Sciences, Bayero University, Kano, Nigeria; 13 International Health and Tropical Medicine, Centre for Tropical Medicine and Global Health, Nuffield Department of Medicine, University of Oxford, Oxford, United Kingdom; 14 Africa Centre of Excellence for Population Health and Policy, Bayero University, Kano, Nigeria; 15 Vanderbilt Institute for Global Health, Vanderbilt University Medical Center, Nashville, Tennessee, United States of America; De Montfort University Faculty of Health and Life Sciences, UNITED KINGDOM OF GREAT BRITAIN AND NORTHERN IRELAND

## Abstract

After decades of control, a nationwide diphtheria outbreak occurred in Nigeria in 2022, with approximately 75% of confirmed cases reported in Kano state, Nigeria. We assessed the spatial distribution of diphtheria cases in Kano state to identify disease clusters/hotspots. We used national surveillance data on 10,085 confirmed cases of diphtheria in Kano state from April 2022 to December 2023, accessed via the Nigerian Centre for Disease Control and Prevention website. Data were converted to CVS format and analyzed for spatial distribution of diphtheria cases using *QGIS-LTR Version 3.34.11*. We found clustering of diphtheria cases in the eight metropolitan Local Government Areas (LGAs) of the state, where health facilities were also clustered. Ungogo LGA had the highest clustering of diphtheria cases but the least clustering of health facilities. This study enhances understanding of the spatial dynamics of diphtheria transmission in Nigeria and provides actionable insights for designing targeted interventions and strategies against hotspots to curb transmission and strengthen preparedness for future epidemics.

## Introduction

Diphtheria is a serious acute infectious disease caused by toxin-producing strains of gram-positive bacteria, *Corynebacterium diphtheriae*, primarily transmitted from person to person through respiratory droplets and contact with infected skin lesions [1]. The pathogenicity and virulence of *C. diphtheriae* are mediated by the production of diphtheria exotoxin, and infection usually results in respiratory (nasal, pharyngeal, or laryngeal) or cutaneous disease [1–3]. Inflammation associated with the diphtheria toxin can result in marked enlargement of the cervical lymph nodes and swelling of surrounding tissues [2,4] and the development of a mucosal pseudo-membrane that can obstruct the airway and and potentially lead to fatal outcomes [5].

Prior to widespread childhood vaccination, it was estimated that approximately 1 million diphtheria cases occurred worldwide, resulting in around 60,000 deaths annually [6]. Following the establishment of the Expanded Programme on Immunization (EPI) in 1974, which initially targeted diphtheria as one of the six deadliest childhood diseases, reported cases declined by >90% between 1980 and 2000 [7]. However, in recent years, diphtheria outbreaks have become more widespread and frequent [8–11]. Although initially limited to vulnerable groups in displaced populations, some outbreaks have also been associated with sub-optimal vaccination coverage and waning of vaccine-induced immunity [8,12]. Sub-optimal coverage and waning immunity can result in successive accumulation of susceptible unvaccinated populations that trigger outbreaks, particularly following immunisation service disruptions that ensued from COVID-19-related restrictions [13,14].

Vaccination coverage has declined globally since the COVID-19 pandemic, with the DTP3 vaccination rate dropping from 86% in 2019 to 81% in 2021, and even lower (56%) in Nigeria, Africa’s most populous country [15–17]. The current diphtheria outbreak started in 2022 and has persisted, and Kano state is the worst afflicted in the country. Between June 2022 and December 2023, a total of 22,293 suspected and 13,387 confirmed diphtheria cases were reported in Nigeria, and Kano state alone accounted for >75% (10,085) of the confirmed cases [18], hence, the reason for the choice of Kano state.

Unlike previous outbreaks of other diseases, where distribution of cases was geographically limited to distinct areas of the state, this diphtheria outbreak is state-wide, with confirmed cases in each of the 44 LGAs of Kano state. Still, the distribution of confirmed cases has not been uniform across the state. We aimed to use data on confirmed cases to understand the epidemiology of this outbreak focusing on their spatial distribution across Local Government Areas (LGAs) within the state, indicating the need for studies that incorporate spatial distribution data alongside incidence and prevalence statistics. Such comprehensive studies will support existing literature to facilitate the development of targeted and effective public health strategies to curb transmission of the disease. We mapped out the geographic distribution of diphtheria cases and identified clusters/hotspots during the 2022/2023 outbreak in Kano state, northern Nigeria.

## Materials and methods

### Study setting

**Fig 1:**
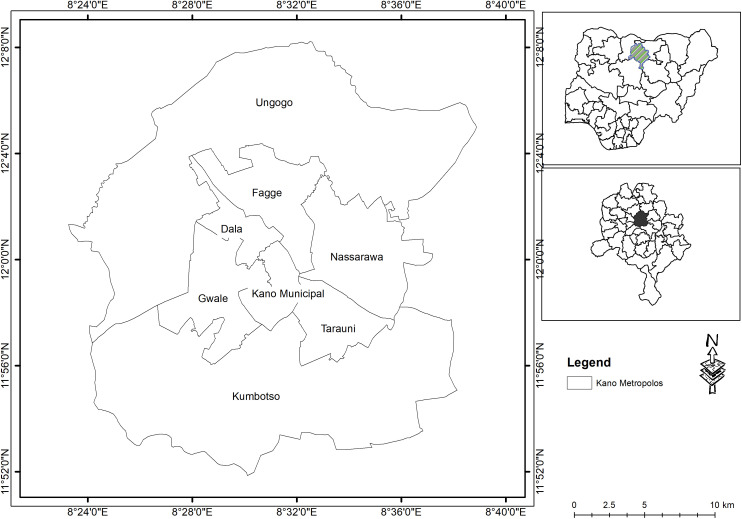
Map of Kano state showing the urban LGAs that comprise Kano Metropolis.

Kano is the most populous state in Nigeria with a 2023 estimated population of about 21 million people [19]. Kano State is home to approximately 10% of Nigeria’s population [19]. Kano has 44 Local Government Areas (LGAs), eight of which are considered urban (Fig 1) while the remaining are rural. The urban LGAs comprising Tarauni, Gwale, Dala, Nassarawa, Kano Municipal, Fagge, Ungogo, and Kumbotso make up about 3% of the state landmass (573 ys 20,131 km^2^), but over a third of the state population. Compared to the average for the state (570 persons per km^2^), these LGAs also have considerably higher population density (average ~20,000 persons per km^2^), ranging from 2,600/ km^2^ in Kumbotso LGA to 45,900/ km^2^ in Dala LGA [20].

### Study design and data source

This study was a descriptive cross-sectional analysis that utilized secondary data. The diphtheria outbreak response was coordinated by the Nigerian Centre for Disease Control and Prevention (NCDC) from April 2022 to December 2023. Reports of cases from various sources, including health facilities, local health departments, and government agencies involved in disease surveillance and control were collated and compiled by NCDC and dataset on all cases is publicly available and accessible via the NCDC website, which we obtained obtained on 8^th^ October 2024 [18]. During the outbreak, all the 36 Nigerian states and the Federal Capital Territory (FCT) were mandated by the NCDC to activate emergency diphtheria response teams in their epidemiology and disease control divisions, with technical support from the NCDC. The response teams were responsible for active surveillance and contact tracing in health facilities and communities. All suspected and confirmed cases in the states were reported to the NCDC through a central dashboard. The analysis included all the 10,085 confirmed cases of diphtheria from Kano State. Data on the location of healthcare facilities in Kano State was obtained from GRID3 which incorporated data from Nigeria Health Facility Registry, National Primary Health care Development Agency and Minimum Standards of Primary Health care in Nigeria [20].

### Data analysis

Data downloaded from the NCDC website were converted into Comma-Separated Values (CVS) format and imported into *QGIS-LTR Version 3.34.11* for analysis and generation of maps. QGIS-LTR is a free and open-source geographic information software available on the official QGIS Project website. The data were then analyzed and presented using choropleth maps to depict the distribution of cases by LGAs and to identify areas with higher incidence rates within the state. We also created heat maps to provide a more granular view of the density of diphtheria cases across LGAs. A shapefile indicating the locations of all health facilities in the state was incorporated into the maps to visualize the spread of health facilities in relation to the identified hotspots.

### Ethical considerations

Ethical clearance was obtained from the Health Research Ethics Committee of the Kano State Ministry of Health (NHREC/17/03/2018 – SHREC/2024/5395). It was a retrospective study of secondary data published online with data fully anonymized before being assessed, and informed consent waived by the ethics committee.

## Results

**Fig 2:**
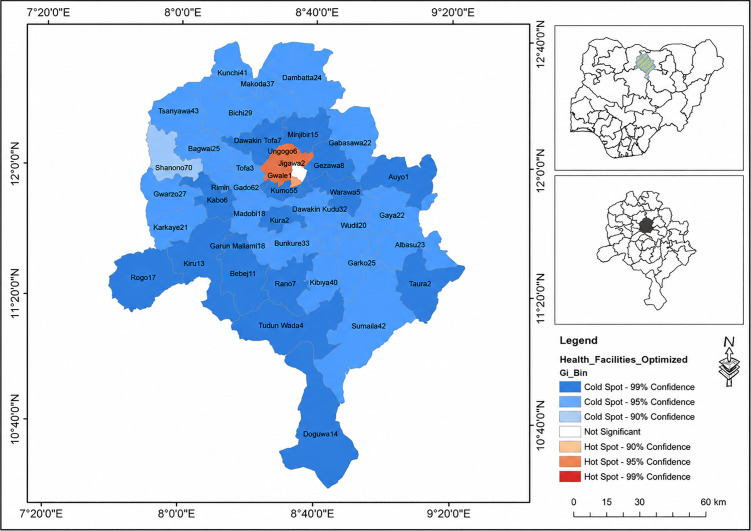
A Choropleth map showing the distribution of diphtheria cases across Local Government Areas in Kano State.

The number of confirmed cases reported varied widely across the LGAs (Fig 2). The metropolitan/urban LGAs were reported identified as hotspots with 99% confidence. However, the majority of the peri-urban LGAs (including Dawakin Tofa, Minjibir, Gezawa, Kumbotso, and Warawa) were identified as cold spots with 99% confidence. Some distant LGAs from the metropolis were also identified as cold spots with 99% confidence, including Rogo, Kiru, Doguwa, Tudun Wada, bebeji, Garun Malam, Rano, Takai and Ajingi. All other LGAs were cold spots with 95% confidence, except Shanono which had 90% confidence. A cold spot indicates that diphtheria cases are significantly underrepresented in an area, while A hotspot indicates that diphtheria cases are significantly represented in an area

**Fig 3:**
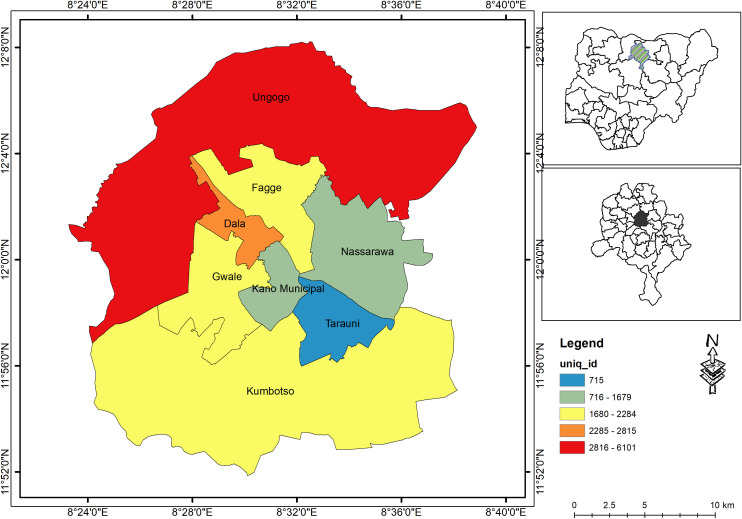
A map showing the distribution of diphtheria cases across the eight urban LGAs making-up Kano Metropolis.

Since all the hotspots for diphtheria cases were within Kano Metropolis, we extracted data for the eight urban LGAs for further analysis (see Fig 1 for map of urban LGAs). Fig 3 is a choropleth map showing the distribution of diphtheria cases across the eight urban LGAs in Kano State. Across these LGAs, reported cases varied widely. Tarauni LGA had the lowest number of cases (<715); followed by Nassarawa and Kano Municipal (716–1679); Gwale, Fagge, and Kumbotso had 1680–2288 cases; Dala had cases in the range of 2285–2815; and Ungogo LGA had the highest number of cases (6,101).

**Fig 4:**
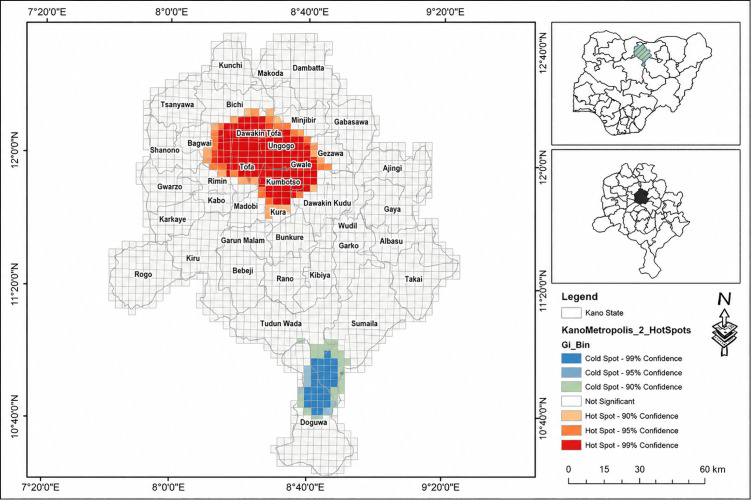
Hotspot analysis of healthcare facilities in Kano State.

The hotspot analysis of healthcare facilities in Kano State (Fig 4) showed a high concentration of healthcare facilities in certain areas, particularly within Kano metropolis. This region is classified as a hotspot with a 99%, 95% and 90% levels of significance. Dawakin Tofa and Tofa LGAs which neighbour the urban LGAs also emerged as hotspots for health facilities. Cold spots were identified in the southern parts of Kano, around Doguwa LGA with 99%, 95% and 90% levels of significance.

A cold spot indicates that healthcare facilities are significantly underrepresented in an area, while a hotspot indicates that healthcare facilities are significantly represented in an area. We found a cold spot in Doguwa LGA, with 95% and 90% significance. The eight metropolitan LGAs were also identified as hotspots for health facilities with 99% and 95% significance. The hotspot analysis also identified several areas where the distribution of healthcare facilities is not significant. These areas neither exhibit significant clustering nor dispersion, suggesting an average distribution of healthcare services.

**Fig 5:**
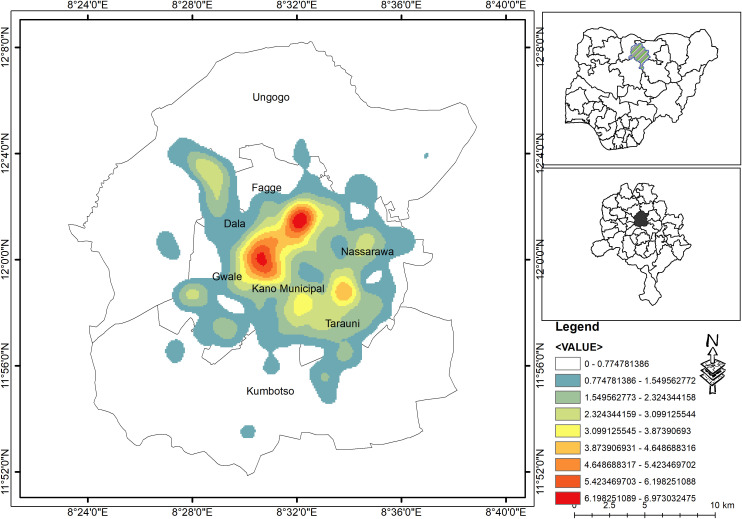
Hotspot analysis of healthcare facilities in the eight LGAs comprising Kano Metropolis.

The distribution of health facilities varied across the eight urban LGAs (Fig 5). We found evidence of clustering for large parts of Fagge, Dala, Kano Municipal, Tarauni, and Gwale. Conversely, for Kumbotso and Ungogo LGAs, we found no evidence of over- or under-representation of health facilities for most parts, except for a few areas where we found evidence of under-representation of health facilities.

**Fig 6:**
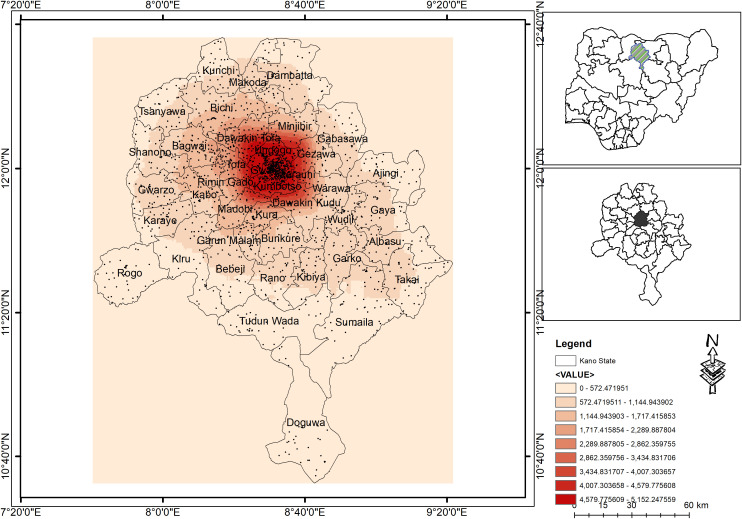
Hotspot map of diphtheria cases with location of healthcare facilities in Kano State.

Fig 6 presents a heatmap of diphtheria cases with a superimposed shapefile indicating locations of healthcare facilities in the State. We found clustering of both diphtheria cases and of healthcare facilities within the eight metropolitan LGAs of the State. This shows that Diphtheria cases were concentrated where health facilities were also concentrated.

## Discussion

We mapped diphtheria cases to visually analyse the spread of diphtheria cases across Kano State. Our analysis indicates a clear clustering of cases in distinct geographic locations of the state, indicating a disproportionate burden of the outbreak within the state. We found clustering of diphtheria cases in Kano Metropolis which make up eight out of the 44 LGAs in Kano state. Kano metropolis makes up about 18.2% of the landmass of all LGAs in the state but is home to about 40% of the state’s population and houses international markets that are patronized by businessmen from across West Africa daily [21].

Diphtheria is transmitted from person-to-person via respiratory droplets or close physical contact. Therefore, high population density in the urban LGAs could facilitate effective contact patterns that enhance more efficient and sustained transmission [22]. We found that the areas with higher concentration of health facilities in rural LGAs reported fewer diphtheria cases and likely less affected by the outbreak. This could be due to the relative isolation/distance of these LGAs from the metropolis. Moreover, the clustering of cases could be partly explained by the high population density and population movement in and out of the city. This can also be explained by findings on diphtheria outbreak in Germany, where cases were mostly associated with migration [23].

In addition to mapping diphtheria cases, the study overlaid a shapefile indicating the location of health facilities across Kano State. This imaging indicated a relationship between the spatial distribution of healthcare facilities and reported incident diphtheria cases. We identified clustering of diphtheria cases in most parts of Kano Metropolis, where healthcare facilities were also clustered. Studies suggest that urban regions, especially in developing countries, tend to have better access to healthcare services, which can positively impact disease control and management [18]. The exception to this finding was Ungogo LGA which had the highest clustering of diphtheria cases but the least clustering of healthcare facilities within the metropolis.

Several factors may have contributed to the resurgence and sustenance of diphtheria and driven the distribution of cases in Kano. First, low uptake of DPT-containing vaccine, has been persistently low in Kano 20%). This poor vaccine uptake leaves large numbers of children unprotected and communities vulnerable to outbreak emergence. Second, vaccine-induced immunity is not life-long and wanes over time. In settings such as ours, where the diphtheria vaccine is only offered in the three primary dose schedules in infancy at 6, 10 and 14 weeks, with no booster, vaccine-induced immunity is likely to wane quickly. Third, vaccination also does not completely protect against colonisation. Therefore, vaccinated persons can still be asymptomatically colonised and can transmit to others, with a strong probability of outbreak in under-vaccinated populations. It is estimated that in outbreak settings, vaccination can only interrupt about 28% of transmission [5]. In Indonesia, 67% of transmission during an outbreak was estimated to be driven by asymptomatic carriers [9]. Fourth, factors that promote effective contacts and transmission, such as overcrowding, poor living conditions and hygiene, are likely to be more prevalent in more populated urban settings [24,25].

Our findings largely indicate the clustering of health facilities and diphtheria cases in the same areas, such that Kano metropolis had the highest clustering for both diphtheria cases and health facilities. This relationship is possibly related to access to diagnosis and ease of reporting from health facilities. Although outbreak surveillance comprised reports from health facilities and communities/households, cases from remote areas with poor geographic access to health facilities. There were a few exceptions. Ungogo had the highest number of reported cases (6,101) but, relative to the other urban LGAs, had a low concentration of health facilities. Conversely, relative to other rural LGAs, Dawakin Tofa and Tofa were hotspots for diphtheria and health facilities. This finding may have two-pronged implications. On the one hand, our findings may indicate that areas with a higher burden of cases have better access to health facilities. On the other hand, there is a possibility that the lower numbers of reported cases in areas with low concentrations of health facilities may be due to under-reporting. Strengthening healthcare infrastructure in these cold spots and addressing the mixed distribution of facilities in other regions will be crucial for improving health equity and ensuring that all residents have access to timely healthcare services during future outbreaks.

Nearly all the diphtheria cases were among children between the ages of 2–14 years, with 87% of them unvaccinated or under-vaccinated [18,22]. A previous analysis of global diphtheria data showed that lower vaccination was associated with having cases aged <15 years, and as vaccine coverage increased the proportion of cases aged ≥15 years also increased [7]. In countries with a high burden, between 66−73% of cases were unvaccinated and only 37% were aged ≥15 years, emphasising the importance of primary vaccine series in protecting the younger population. In contrast, in countries with sporadic cases, 32% were unvaccinated, and 66% were aged 15 years, indicating the role of waning immunity and the importance of booster doses in older ages. We hypothesize that low diphtheria vaccine coverage is likely to be the main driver for the outbreak, particularly following the breakdown of health systems and health commodities supply chain following the COVID-19 pandemic [7,9]. This may have further decreased herd immunity that prompted the outbreak. Additionally, changes in contact patterns during the COVID-19 movement restrictions may have facilitated close contact and enhanced transmission. This can also be explained by the post-COVID-19 diphtheria outbreak in high-income countries like Germany and Poland between 2022–2023 [23]. Another possible explanation is the prevalence of asymptomatic colonisation, particularly in settings that favour transmission – high population density, overcrowding, and poor hygiene [26]. The bulk of cases in children beyond the vaccine-eligible age group may be indicative of lower effective contacts in very young children and the need for booster doses in older persons [7].

This study is strengthened by the availability of comprehensive diphtheria national surveillance data and geographic coordinates for all health facilities in Kano State, which collectively made the analysis robust. However, our analysis is limited by not having geographic coordinates for all diphtheria cases, therefore, all analyses were performed at the LGA level. We were, therefore, unable to identify smaller units of hotspots, perform nearest neighbor analysis and spatial autocorrelation, or assess the distance to healthcare facilities.

This study fills an important gap in understanding the spatial dynamics of diphtheria transmission in Kano State. The analysis offers valuable insights for public health officials to design targeted interventions and strategies aimed at disease hotspots. These insights can help curb the spread of the disease and enhance preparedness for future epidemics by supporting targeted vaccination campaigns and strengthening routine immunization programs to boost population immunity. Identifying vulnerable areas with under-representation of health facilities will assist in addressing inequity that may result from the health facility distribution disparities. The distribution of diphtheria cases indicates the importance of understanding contexts to guide targeted and appropriate outbreak response interventions. Addressing these disparities is essential for preparing for future outbreaks and similar public health challenges in Northern Nigeria, and the findings can be extrapolated in other similar settings
